# Machine learning approach to needle insertion site identification for spinal anesthesia in obese patients

**DOI:** 10.1186/s12871-021-01466-8

**Published:** 2021-10-18

**Authors:** Jason Ju In Chan, Jun Ma, Yusong Leng, Kok Kiong Tan, Chin Wen Tan, Rehena Sultana, Alex Tiong Heng Sia, Ban Leong Sng

**Affiliations:** 1 Department of Women’s Anesthesia, KK Women’s and Children’s Hospital, 100 Bukit Timah Road, Singapore, 229899 Singapore; 2 Anesthesiology and Perioperative Sciences Academic Clinical Program, Duke-NUS Medical School, 8 College Road, Singapore, Singapore; 3 Department of Electrical and Computer Engineering, Faculty of Engineering, National University of Singapore, Singapore, Singapore; 4 Center for Quantitative Medicine, Duke-NUS Medical School, 8 College Road, Singapore, Singapore

**Keywords:** Neuraxial anesthesia, Spinal, Automated, Ultrasound

## Abstract

**Background:**

Ultrasonography for neuraxial anesthesia is increasingly being used to identify spinal structures and the identification of correct point of needle insertion to improve procedural success, in particular in obesity. We developed an ultrasound-guided automated spinal landmark identification program to assist anesthetists on spinal needle insertion point with a graphical user interface for spinal anesthesia.

**Methods:**

Forty-eight obese patients requiring spinal anesthesia for Cesarean section were recruited in this prospective cohort study. We utilized a developed machine learning algorithm to determine the needle insertion point using automated spinal landmark ultrasound imaging of the lumbar spine identifying the L3/4 interspinous space (longitudinal view) and the posterior complex of dura mater (transverse view). The demographic and clinical characteristics were also recorded.

**Results:**

The first attempt success rate for spinal anesthesia was 79.1% (38/48) (95%CI 65.0 - 89.5%), followed by successful second attempt of 12.5% (6/48), third attempt of 4.2% (2/48) and 4th attempt (4.2% or 2/48). The scanning duration of L3/4 interspinous space and the posterior complex were 21.0 [IQR: 17.0, 32.0] secs and 11.0 [IQR: 5.0, 22.0] secs respectively. There is good correlation between the program recorded depth of the skin to posterior complex and clinician measured depth (*r* = 0.915).

**Conclusions:**

The automated spinal landmark identification program is able to provide assistance to needle insertion point identification in obese patients. There is good correlation between program recorded and clinician measured depth of the skin to posterior complex of dura mater. Future research may involve imaging algorithm improvement to assist with needle insertion guidance during neuraxial anesthesia.

**Trial registration:**

This study was registered on clinicaltrials.gov registry (NCT03687411) on 22 Aug 2018.

## Introduction

Neuraxial procedures are commonly performed for a wide range of therapeutic and diagnostic indications. These include neuraxial anesthesia for surgery, labour epidural analgesia, neuraxial steroid injections and diagnostic lumbar punctures [1]. However, the current method of palpation to locate the point of needle insertion is known to be associated with a significant failure rate (27 to 32%) [2, 3]. The administration of spinal anesthesia at an inappropriately high intervertebral level may result in permanent neurological injury. Multiple puncture attempts may increase the risk of complications such as post-dural headache, paraesthesia and spinal hematoma [4, 5]. The prevalence of obesity in pregnant women is increasing, ranging from 5.5 to 38.3% [6]. Neuraxial anesthesia in obesity is anatomically more challenging due to the difficulty with palpating spinal landmarks.

Neuraxial ultrasonography has become increasingly popular for neuraxial space identification [7–9], and has since been recommended for clinical use [9, 10]. It is a safe and effective technique, with increasing use as an auxiliary over physical palpation to improve the overall success rate of neuraxial procedures and to reduce insertion attempts. Geng et al. reported a first attempt success rate of neuraxial blocks using ultrasound of 68.4% [11]. A recent meta-analysis demonstrated that ultrasound imaging could reduce the risk of failed or traumatic lumbar punctures and epidural catheterization, as well as the number of insertion attempts [9].

Neuraxial ultrasonography in obese patients is limited by the considerable scanning depth, the skill for acquiring good images and the image interpretation. The steep learning curve and difficulty of pattern recognition of spinal structures can be challenging to even experienced operators, especially when difficult spinal anatomy is present [12–14].

We have previously developed an ultrasound-based guidance program to determine the optimal insertion site and angle for neuraxial procedures [15–18]. However, only patients with body mass index (BMI) below 30 kg/m^2^ were recruited. In this study, we refined the program with image processing techniques and machine learning algorithm to be used in obese patients (BMI > 30 kg/m^2^) undergoing spinal anesthesia.

The primary aim was to evaluate the first attempt success rate of spinal anesthesia in obese patients, using landmarks obtained from an improved automated spinal landmark identification algorithm. The primary hypothesis of the study was that the automated spinal landmark identification algorithm using an improved image processing system would achieve greater than 68.4% [11] first attempt success rate of spinal anesthesia in patients with BMI greater than 30 kg/m^2^.

## Methods

### Patient recruitment

This study was approved by the SingHealth Centralized Institutional Review Board, Singapore (SingHealth CIRB Ref: 2018/2021), and registered on Clinicaltrials.gov (NCT03687411). The study period was between May 2018 and Feb 2019. We recruited female patients above 21 years old who required spinal anesthesia for Cesarean section, with a BMI of more than 30 kg/m^2^. The exclusion criteria were a history of scoliosis or spinal instrumentation, allergy to ultrasound transmission gel, and patients with visible wound or injury to the lumbar spine. Written informed consent was obtained from every patient before any study procedures. This manuscript adheres to the applicable Strengthening the Reporting of Observational Studies in Epidemiology (STROBE) guidelines, and conducted in accordance with the Declaration of Helsinki.

**Fig. 1:**
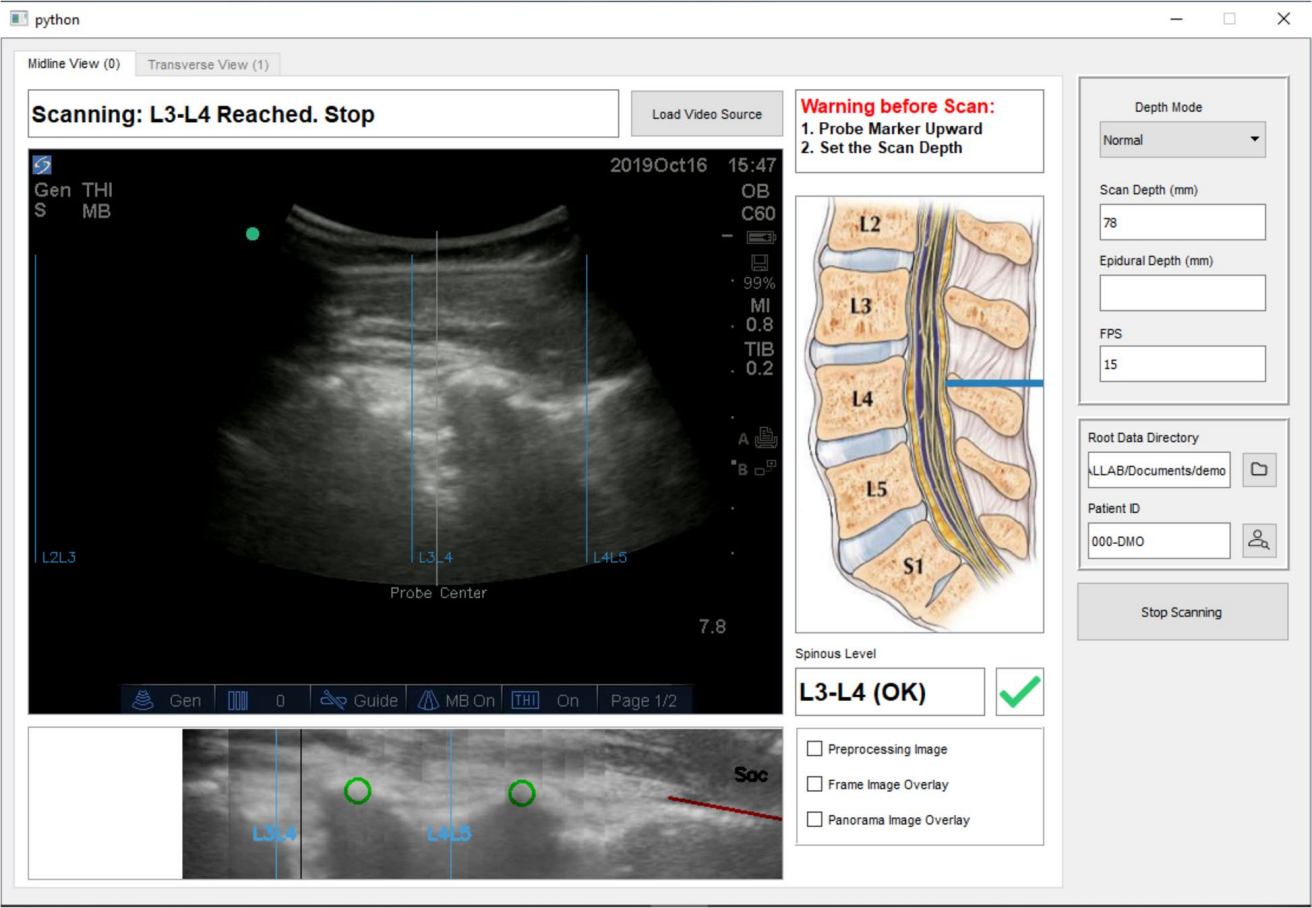
GUI for the longitudinal view

The patient assumed a seated position with lower back exposed. Ultrasound gel would be applied to the lower back lumbar spine and the investigator would place the ultrasound curvilinear probe around the sacral region for the longitudinal view. The graphical user interface (GUI) of the software, integrated with the ultrasound machine, guided the investigator to first identify the sacrum as a hyperdense line (reflected as a marked red line as shown in Fig. 1). The investigator then moved the probe in a steady vertical upward longitudinal direction of the lumbar spine. The system displayed the centre of the probe as a grey vertical line and the interspinous spaces as blue vertical lines labelled according to the space identified. Laminae were reflected as green circles in the GUI. Upon identification of the L3/4 interspinous space, the investigator would make skin markings along the midpoint of the probe using a surgical skin marker.

**Fig. 2:**
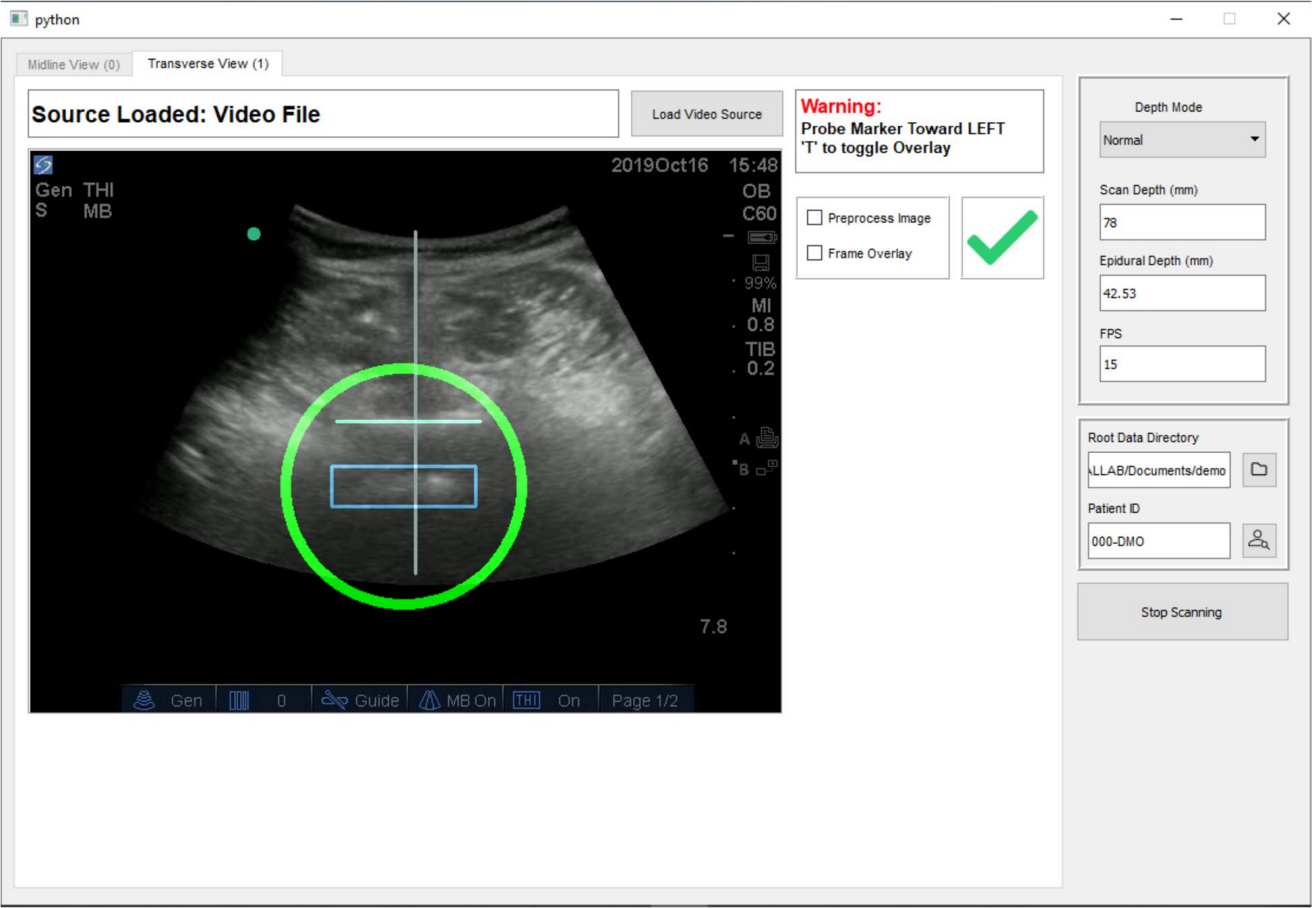
GUI for the transverse view

For the transverse view, the probe was turned 90 degrees clockwise to a horizontal position along the previously marked line at level of L3/4, with minimal rotational movements to obtain the best view. The software program guided the investigator to the best view by indicating a green tick on the screen (Fig. 2). The green tick would not appear if the view required by the software program was not obtained. The software would signal when the optimal position and orientation of the posterior complex of dura mater was identified. The investigator would then mark a vertical line on the skin at the midline of probe, using a surgical skin marker.

After this scan sequence was completed, the anesthetist in the operating theatre attempted spinal anesthesia needle insertion based on the needle entry insertion point and angle identified by the program. The first attempt was done without traditional palpation technique. However, if dural puncture was not obtained at the first attempt, subsequent attempts could include the use of traditional palpation of spinal landmarks. The number of spinal attempts was defined as the number of spinal needle insertion points on the skin, which was recorded until the successful attempt was achieved. Patient demographic data including age, race, weight, height, and history of spine disorders were also recorded.

The duration taken to identify the L3/4 interspinous space (longitudinal view) and the posterior complex (transverse view) were also recorded. All the identified landmarks in the longitudinal view and the transverse view were validated by the clinician investigators to validate the program after recruitment. The distance from skin to posterior complex was also measured by the program. This was followed by the reading of the recorded scans by an experienced clinician investigator, blinded to the recorded images and videos by using study numbers, to determine the distance from skin to posterior complex from the scans. Congruency between the distance as measured by the program and by the clinician investigator was then determined. In this study, the scans were done only by the principal investigator and co-investigators who are attending consultant anesthetists. However, the spinal needle insertions were done by either attending consultant anesthetists or anesthetic trainees who were assigned to the operating theatre.

### Ultrasound and software set-up

**Fig. 3:**
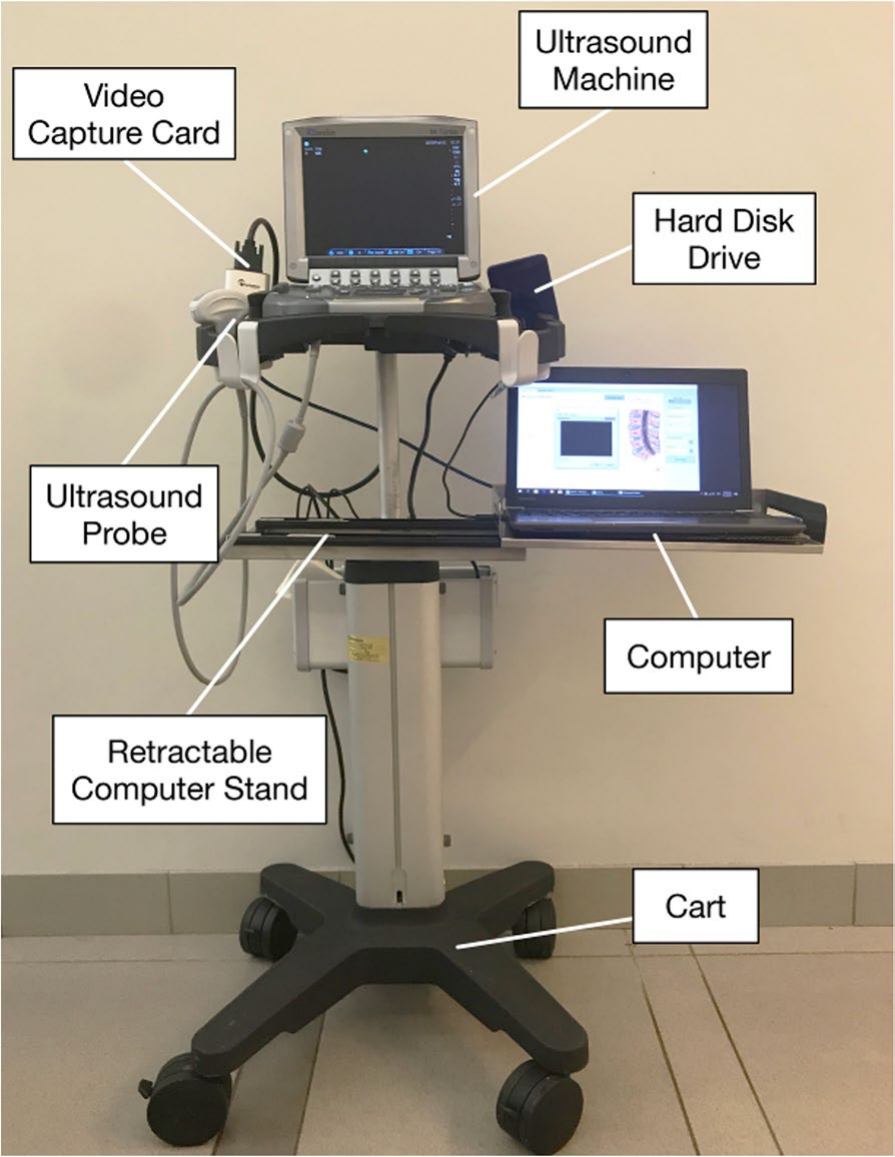
System setup of the automated ultrasound-guided spinal landmark identification program

A SonoSite M-Turbo ultrasound machine (SonoSite, Bothell, USA) was connected to a Dell Latitude E5450 Laptop to stream real-time video through an Epiphan DVI2USB 3.0 video capture card (Epiphan, Ottawa, Canada). A retractable computer stand was fabricated and installed on the ultrasound machine’s cart, which allowed the anesthetists to move the system efficiently in the operating theatres (Fig. 3). The chosen ultrasound machine could produce image frames of 640 × 480 pixels at a rate of 15 frames per second, whereas the video capture card was able to perform video conversion at a maximum resolution of 1920 × 1200 pixels with a maximum rate of 30 frames per second. In the clinical setup, raw video signals from the ultrasound machine would be first sent to the video capture card, after which the converted images would be sent to the laptop in real time study. All the videos during the scanning were automatically saved in an encrypted hard disk with the study number only, to maintain patient confidentiality.

Prior to the study, the clinician investigators were trained to use the ultrasound setup and program to determine the optimal insertion point and angle prior to subject recruitment. To allow automated spinal landmark identification, offline machine learning algorithm training was conducted upon the historical data collected from previous studies at our research group [19]. After the scan of each patient, saved videos and images were used for post-processing purpose, where fine-tuning of the program was done. These changes were then incorporated as part of the refined program.

Improvements in the software program included automated sacrum identification, interspinous space detection, level counting, and panorama stitching in the longitudinal plane and neuraxial space identification in the transverse plane. All these functions are integrated into the GUI, where the ultrasound images, identification results, and all the relevant operation instructions are displayed in real-time.

Especially, to cater for patients with different BMIs, two modes were provided with different scanning depths, which are named “Normal Mode” and “Obese Mode” in the GUI. In the GUI, the scanning depth was set as 78 mm in “Normal Mode”, 92 mm in “Obese Mode”. The user could make the selection based on the image quality and structures acquired on the ultrasound machine.

### Sample size and statistical analysis

The planned sample size for the primary aim of this study of at least 43 patients was calculated based on the following assumptions: expected success rate of 68.4% [11] at first attempt of spinal needle insertion using the automated spinal landmark identification system among obese patients with BMI > 30 kg/m^2^ with 95% confidence interval of width 27.0% i.e. success rate at first attempt will lie between 53.5 and 80.3%. After adjusting for 10% failure rate to obtain successful imaging, planned sample size was 48.

The primary outcome, success at first attempt of spinal needle insertion, was treated as binary data with status either “*yes*” or “*no*”. Proportion of success rates was expressed as proportion with corresponding 95% confidence interval (CI) using exact confidence interval approach. Demographic and imaging data were summarized based on the status of success at first attempt. Continuous variables were summarized as mean ± standard deviation (SD) or median [interquartile range (IQR)], whichever appropriate, while categorical variables were summarized as frequency (proportions). Pearson’s correlational coefficient and Cronbach’s Alpha were used to assess the internal reliability of program recorded depth of skin to posterior complex of dura mater and the clinician measured depth. All statistical tests performed were two – sided and *p* – value < 0.05 was considered as statistical significance. All statistical analyses were performed in SAS version 9.4 software (SAS Institute, Cary, North Carolina).

## Results

From May 2018 to February 2019, 50 patients were recruited for this study. However, two patients were excluded due to patient decision for vaginal birth after Cesarean section after recruitment. The mean age of patients was 32.3 ± 4.8 (ranged 22 - 44) years, with an average BMI of 35.0 ± 4.5 kg/m^2^.

**Fig. 4:**
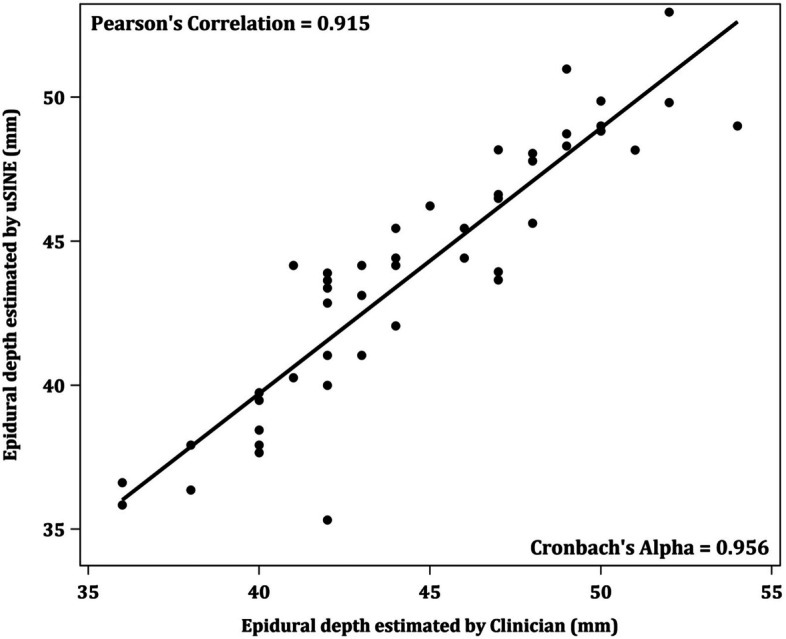
Pearson’s correlation and Cronbach’s alpha between program recorded depth of the skin to posterior complex and the clinician measured depth

We further grouped the patients according to the successful dural puncture at first attempt based on the needle insertion site and angle automatically determined by the program (Table 1). Thirty-eight patients (79.1, 95% CI 65.0 - 89.5%) had successful dural puncture at first attempt (‘First-attempt group’), whereas the rest were successful only after two (*n* = 6 or 12.5%), three (*n* = 2 or 4.2%), and four (*n* = 2 or 4.2%) puncture attempts (‘Not at first attempt group’). The BMIs between successful first attempt group and ‘not at first attempt group’ did not show significant difference. The scanning duration of L3/4 interspinous space and the posterior complex were 21.0 [IQR: 17.0, 32.0] secs and 11.0 [IQR: 5.0, 22.0] secs respectively. With that, the average number of puncture attempts was 1.3, with a standard deviation of 0.75. The Pearson’s correlation coefficient and Cronbach’s alpha between the program recorded depth of the skin to posterior complex and the clinician measured depth was 0.915 and 0.956, respectively (Fig. 4).

**Table 1:** Demographic and clinical characteristics based on the success rates of the spinal insertion

**Variables**	**Success at**		***P* – value**
	**First attempt (*n* = 38)**	**Not at first attempt (*n* = 10)**	
Race, n (%)			0.1630
Chinese	15 (39.5)	1 (10.0)	–
Malay	11 (28.9)	6 (60.0)	–
Indian	8 (21.1)	3 (30.0)	–
Others	4 (10.5)	0 (0.0)	–
Age (years), mean ± SD	32.3 ± 4.9	32.0 ± 4.6	0.9493
Weight (kg), mean ± SD	88.5 ± 15.3	88.1 ± 9.1	0.7128
BMI (kg/m^2^), mean ± SD	35.0 ± 4.8	34.9 ± 3.4	0.6476
Level of scan operator Consultant, n (%)	38/38 (100)	10/10 (100)	–
Skin to posterior complex depth (mm), mean (SD)	43.7 ± 4.7	45.2 ± 3.6	0.1922
Use of ‘Obese’ Mode, n (%)	7/38 (18.4)	3/10 (30.0)	0.4143
Midline scanning duration (secs), median [IQR]	21.0 [16.0, 29.0]	25.0 [18.0, 37.0]	0.4847
Transverse scanning duration (secs), median [IQR]	11.0 [5.0, 21.0]	9.0 [5.0, 22.0]	0.8758

*P* values are based on fisher’s exact test for categorical variable and two sample t-test or Mann-Whitney U - test for continuous variables

## Discussion

Obstetric patients with obesity could be scanned successfully by using this developed program. Anatomical landmarks were identified in both longitudinal view and transverse view in the majority of cases. We also observed a good correlation between the program recorded depth of skin to posterior complex of dura mater and the clinician measured depth.

Shaylor et al. achieved an overall success rate of 90.5% at first attempt for ultrasound guided neuraxial block [20]. However, their study had one experienced operator performing both ultrasound and spinal blocks. In contrast, our study had multiple operators (trained consultants performing the ultrasound and trainee anesthetists performing the spinals). Inter-user variability for ultrasound image acquisition and difference in experience of anesthetists performing the spinals, could contribute to the lower first attempt success rate. The successful first attempt rate reported in this study (79.1%) is higher than one described by Hood and Dewan (42%) whereby palpation directed surface landmarking was employed in obese patients [21]. Our success rate is also higher than studies by Geng et al. (68.4% [11]) and Chin et al. (65% [22]) whereby ultrasound-guided spinal anesthesia was used. This could reflect clinical practice success rates from an obstetric anesthesia training centre.

The challenge for neuraxial procedure lie in the determination of spinal level of lumbar spine and midline position for needle insertion. Pregnant women who are obese could have a longer distance to the neuraxial space with often difficulty palpating the spinal landmarks due to the increased fat tissue [6]. Pregnancy is also associated with weight gain, tissue oedema and increased lordosis which can make palpation and identification of surface landmarks more difficult. These changes may also increase the risk for inadvertent dural puncture [23].

The use of ultrasound to assist neuraxial block placement increases the initial procedural success rate performed by a junior trainee and facilitates reaching a competent skill level earlier [24, 25]. This program combines all the required functions for identifying the optimal location and orientation for needle entry for junior trainees using the intuitive GUI and workflow in this ultrasound program.

Grau et al. studied the influence of tissue alterations of pregnancy on epidural technique and graded how well the main anatomical landmarks were visualized by ultrasonography [26]. In our study, we found a good correlation between the program measured and clinician measured depth of skin to posterior complex distance. Future studies should be done to validate the findings of this program.

Ultrasound imaging could be useful for patients with obesity, abnormal spine, scoliosis and previous spinal surgery [22]. Chin et al. reported that neuraxial anesthesia needle insertion first attempt success rate was improved from 32% (palpation technique) to 65% (ultrasound imaging) in patients with abnormal spinal anatomy [22]. Our program could be further developed in patients with more complex spinal conditions.

There were several limitations in this study. Firstly, we aimed to recruit obese patients with BMI above 30 kg/m^2^. As compared with our previous study on obstetric women with BMI below 30 kg/m^2^ [19], the difference in BMI resulted in a lower first attempt success rate, probably due to the lower image quality of ultrasound images in obese patients [26–28]. We were also limited by the high sensitivity required to produce ultrasound images of good quality in the proposed image processing program. Obese patients may have longer distances to the neuraxial space and increased fat tissue tends to downgrade the image quality. Although the program was able to identify both the anterior and posterior complexes, the deeper anterior complex was not visualised in several images in obese patients and hence was not included in the analysis. This program was also not designed or validated for complex spinal anatomy, pediatric patients and geriatric patients as the offline training did not take these cases in the training set. Some technical challenges were faced during the scanning, such as unstable connections between the ultrasound machine and the laptop. Variations to the brightness in the acquired ultrasound images also led to some difficulties in landmark identification especially in frames with low brightness.

## Conclusion

As a compact addition to current ultrasound systems, we have shown that the developed program may be used for assisting anesthesiologists in neuraxial anesthesia procedure, which allows smooth integration into the current clinical workflow. In conclusion, the study found that this novel system resulted in high accuracy of spinal anesthesia with acceptable procedural scan time. The system will also reduce the occurrence of complications and unnecessary discomfort induced by multiple puncture attempts, translating to better patient experience.

## Data Availability

All the raw data in Table 1 and results section is available in Figshare, an online open access repository (10.6084/m9.figshare.14870076.v1). No further administrative permission is required for the access of raw data as indicated in SingHealth Cluster Policies SHS-RSH-OOR-CWP-202 (version 1.0) and SHS-RSH-OOR-CWP-203 (version 1.0).We are not able to provide the data as the videos are only readable within the program in encrypted format. Queries on the program may be addressed by contacting the corresponding author.

## Abbreviations

BMI: Body mass index
CI: Confidence interval
GUI: Graphical user interface
IQR: Interquartile range
SD: Standard deviation
STROBE: Strengthening the Reporting of Observational Studies in Epidemiology

## References

[1] Osterman MJ, Martin JA (2011). Epidural and spinal anesthesia use during labor: 27-state reporting area, 2008. Natl Vital Stat Rep.

[2] Hermanides J (2012). Failed epidural: causes and management. Br J Anaesth.

[3] Ready LB (1999). Acute pain: lessons learned from 25,000 patients. Reg Anesth Pain Med.

[4] Sawyer RJ (2000). Peripheral nerve injuries associated with anaesthesia. Anaesthesia.

[5] Paech MJ, Godkin R, Webster S (1998). Complications of obstetric epidural analgesia and anaesthesia: a prospective analysis of 10,995 cases. Int J Obstet Anesth.

[6] Saravanakumar K, Rao SG, Cooper GM (2006). Obesity and obstetric anaesthesia. Anaesthesia.

[7] Whitty R, Moore M, Macarthur A (2008). Identification of the lumbar interspinous spaces: palpation versus ultrasound. Anesth Analg.

[8] Ecimovic P, Loughrey JP (2010). Ultrasound in obstetric anaesthesia: a review of current applications. Int J Obstet Anesth.

[9] Shaikh F (2013). Ultrasound imaging for lumbar punctures and epidural catheterisations: systematic review and meta-analysis. BMJ.

[10] Ultrasound guided catheterization of the epidural space: understanding NICE guidance. Interventional Procedures Guidance [IPG249] 2008. Available from: https://www.nice.org.uk/guidance/ipg249/chapter/1-Guidance. Cited 2019 15 October.

[11] Geng J (2016). Ultrasound imaging increases first-attempt success rate of neuraxial block in elderly patients. Zhonghua Yi Xue Za Zhi.

[12] Margarido CB (2010). Anesthesiologists’ learning curves for ultrasound assessment of the lumbar spine. Can J Anaesth.

[13] Deacon AJ, Melhuishi NS, Terblanche NC (2014). CUSUM method for construction of trainee spinal ultrasound learning curves following standardised teaching. Anaesth Intensive Care.

[14] Halpern SH (2010). The use of ultrasound for lumbar spinous process identification: a pilot study. Can J Anaesth.

[15] Kerby B (2008). Automatic identification of lumbar level with ultrasound. Annu Int Conf IEEE Eng Med Biol Soc.

[16] Yu S (2014). Feature extraction and classification for ultrasound images of lumbar spine with support vector machine. Annu Int Conf IEEE Eng Med Biol Soc.

[17] Yusong L (2016). Development of a real-time lumbar ultrasound image processing system for epidural needle entry site localization. Annu Int Conf IEEE Eng Med Biol Soc.

[18] Ikhsan M (2017). Gabor-based automatic spinal level identification in ultrasound. Annu Int Conf IEEE Eng Med Biol Soc.

[19] Oh TT (2019). A novel approach to neuraxial anesthesia: application of an automated ultrasound spinal landmark identification. BMC Anesthesiol.

[20] Shaylor R (2016). High success rates using ultrasound for neuraxial block in obese patients. Isr Med Assoc J.

[21] Hood DD, Dewan DM (1993). Anesthetic and obstetric outcome in morbidly obese parturients. Anesthesiology.

[22] Chin KJ (2011). Ultrasound imaging facilitates spinal anesthesia in adults with difficult surface anatomic landmarks. Anesthesiology.

[23] Lee A (2014). Ultrasound in obstetric anesthesia. Semin Perinatol.

[24] Grau T (2003). Ultrasound imaging improves learning curves in obstetric epidural anesthesia: a preliminary study. Can J Anaesth.

[25] Vallejo MC (2010). Ultrasound decreases the failed labor epidural rate in resident trainees. Int J Obstet Anesth.

[26] Grau T (2001). The lumbar epidural space in pregnancy: visualization by ultrasonography. Br J Anaesth.

[27] Mace HS, Paech MJ, McDonnell NJ (2011). Obesity and obstetric anaesthesia. Anaesth Intensive Care.

[28] Uppot RN (2007). Impact of obesity on medical imaging and image-guided intervention. AJR Am J Roentgenol.

